# The Hotel-Dieu 50 Years Ago

**Published:** 1844-08-10

**Authors:** 


THE HOTEL-DIEU 50 YEARS AGO.

In 1786, diseases and infirmities of every nature and description were treated in the Hôtel-Dieu. The patient who arrived was placed in the bed and sheets of the patient who had just died, affected with the small-pox, it might be, or some other disease, complicated with itch. Two insane patients were always laid in one bed. (Think of two madmen under the same coverlid!) In the ward exclusively reserved for small-pox patients, there were often six adults, or eight children, laid in a bed less than 5 feet in width. On the female side there was no separate ward for small-pox; the patient labouring under a trifling fever had perchance for her bed-fellow a woman in the height of this dreadful malady! The patients were laid head and heels, the feet of one being in contact with the shoulders of another; and as four, and even six, were laid in a bed 4 ft. 8 in. English in width, each could have had no more than from nine to ten inches for his portion. But as a man of ordinary stature is eighteen or nineteen inches across the shoulders, it was, of course, impossible for the unfortunate people to be all in bed together, unless laid upon their sides and completely motionless. No one could move, or attempt to turn without disturbing or waking those on either side of him; it was therefore usual for them to have the bed by turns, two or three sitting up, as long as they were able to do so, two or three lying down. And then the food and the physic destined for one was frequently given to another; the dying were frequently heard to curse the kindest hand that sought to soothe them; life in such wretchedness being less desirable or desired than death. Dreadful to relate, dreadful to think of, those who died in the night always remained mingled with the living till morning! By and by an *improvement*—the first that was made in the Hôtel-dieu—was introduced: the testers were fitted up as beds, and the patients were placed in tiers one over the other! The heart sickens, the understanding shrinks from, the spirit is confounded, with the contemplation of so much misery; and yet this hospital existed fifty years ago, in the midst of a capital, the centre of the arts, of learning, of polished manners, beside the palace of an opulent archbishop, at the door of a magnificent cathedral! It was in such circumstances that the wretched sufferer from disease, under the deceitful mask of charity, was tempted to his destruction; for the diseases were of twice the duration at the Hôtel-Dieu that they were at La Charité, and the mortality was twice as great; all who were trepanned died, and the women in childbed fell victims in a frightful proportion.

At whose door lies the charge of this barbarous organization? At that of the medical staff? No, no; far otherwise. By an inconceivable anomaly the medical officers of hospitals have never had more than a very secondary influence over their administration. No, but at that of the vulgar omnipotence of routine and of ignorance. "The Hôtel-Dieu," said the sage Bailly, "has perhaps existed since the seventh century; and if this hospital be the worst of all, it is only so because it is the oldest. There has still been a leaning to keep things as they were in the beginning; constancy in every thing has appeared a duty; hence the difficulty that aught new, however useful, finds in gaining an entrance. All reform was extremely difficult; the administration that must be convinced was numerous; it was an enormous mass to move." But this magnitude of the mass, the difficulties of the enterprise, did not daunt the members of the Academy of Sciences, with Bailly, Laplace, and Lavoisier, at their head. Every patient is now laid in his own bed; and that he is so is mainly due to the persevering unanimity, the courageous efforts, of the Academy of Sciences. The poor man ought to be aware of this: he will assuredly not forget it. Happy, my friends, happy the Academy that can crown itself with such dear recollections.—*M. Arago, in his Elogé de Bailly.*

[How sad to think that two of these noble natures fell victims to the fearful madness of the French Revolution!—Bailly and Lavoisier are said to have been carried to the slaughter in the same cart!]—*London Med. Gaz.*

